# Combination Effect of Three Main Constituents From *Sarcandra glabra* Inhibits Oxidative Stress in the Mice Following Acute Lung Injury: A Role of MAPK-NF-κB Pathway

**DOI:** 10.3389/fphar.2020.580064

**Published:** 2021-01-11

**Authors:** Chun-Ping Liu, Jian-Xing Liu, Jiangyong Gu, Fang Liu, Jin-Hua Li, Bin-Yang, Yuan-Zheng, Jie-Li, Shou-hai Wu, Qing-he Wu, Xian-Zhang, Long-Mei Li, Hai-Long Yang, Lei Wang, Xiong Li

**Affiliations:** ^1^ The Second Affiliated Hospital of Guangzhou University of Chinese Medicine, Guangzhou, China; ^2^ Dongguan and Guangzhou University of Chinese Medicine Cooperative Academy of Mathematical Engineering for Chinese Medicine, Dongguan, China; ^3^ Research Center of Integrative Medicine, School of Basic Medical Science, Guangzhou University of Chinese Medicine, Guangzhou, China; ^4^ Institute of Tropical Medicine, Science and Technology Innovation Center, Guangzhou University of Chinese Medicine, Guangzhou, China; ^5^ Guangzhou Medical University School of Basic Medicine, Guangzhou, China

**Keywords:** *Sarcandra glabra*, chlorogenic acid, rosmarinic acid, isofraxidin, acute lung injury, MAPK-NF-kB 3

## Abstract

Caffeoylquinic acids, coumarins and dicaffeoyl derivatives are considered to be three kinds of the most abundant bioactive components in *Sarcandra glabra*, an anti-inflammatory herb mainly found in Southern Asia. The combined anti-inflammatory effect of three typical constituents C + R + I (chlorogenic acid + rosmarinic acid + isofraxidin) from this plant has been investigated. The result implies that targeting the MAPK-NF-κB pathway would be one of the major mechanisms involved, using LPS stimulated RAW 264.7 cells as *in vitro* model and LPS-induced acute lung injury in mice as *in vivo* model. C + R + I can significantly suppress the levels of nitric oxide (NO), pro-inflammatory cytokines, and inhibit iNOS and COX-2 expression in LPS-treated RAW264.7 macrophage cells. Western blot analysis showed that C + R + I suppressed phosphorylation of NF-κB and MAPK, including phosphorylation of p65-NF-κB, IKB, ERK, JNK and P38. Besides, C + R + I suppressed MPO protein expression, but promoted SOD and HO-1 expression, and the related targets for C, R, and I were also predicted by molecular docking. This indicated that C + R + I could alleviate oxidative stress induced by LPS, which were further verified in the *in vivo* model of mice with acute lung injury through the measurement of corresponding inflammatory mediators and the analysis of immunehistochemistry.

## Introduction

Acute lung injury (ALI) or acute respiratory distress syndrome (ARDS) is a central complication during the pathological process in the patients with severe coronary virus disease 2019 (COVID-19). It is found that the patients diagnosed with ALI/ARDS have poorer prognosis and higher mortality (Li et al., 2020; van de Veerdonk et al., 2020). ALI and its severe form, ARDS, can lead to rapid loss of lung function, characterized by overwhelming inflammatory responses in the lung (Rebetz and Semple, 2018). Lipopolysaccharide (LPS) from gram-negative bacteria can promote inflammatory responses to mammalians, and has been chosen as the inflammatory inducer to develop the experimental model of ALI/ARDS (Chen et al., 2018). As indicated by previous research, MAPK-NF-κB signaling is a key pathway which is involved in the regulation of ALI/ARDS (Lv et al., 2017; de Oliveira et al., 2019). Likewise, free radicals can trigger inflammatory signaling, resulting in an increased level of circulating pro-inflammatory cytokines, which are known to induce ALI/ARDS (Zielińska et al., 2017). Thus, the treatments aimed at the inhibition of NF-κB and MAPKs, as well as the direct inhibition of oxidative stress, may have potential therapeutic advantages in curing ALI/ARDS. Anti-inflammatory active ingredients have a widespread presence in Chinese medicine, which have exhibited great therapeutic effect through multi-component and multi-target approach. The study of the main anti-inflammatory ingredients and their synergistic mechanism will help us to understand the nature of the role of Chinese medicine (Chen et al., 2019a; Chen et al., 2019b).

*Sarcandra glabra* (Thunb) Nakai (*S. glabra*) is an herbal medicine distributed in Southern Asia. According to Chinese Pharmacopoeia, the entire plant of *S. glabra* and its extract can be used to treat respiratory tract inflammation related diseases such as acute respiratory infections, pneumonia, and so on (National Commission of Chinese Pharmacopoeia, 2015). Furthermore, this herb has also been recommended to prevent and treat COVID-19 by the Chinese government (Liang et al., 2020). Several types of components in *S. glabra* have been purified and identified by the LC-MS technique (Li et al., 2011). 17 representative compounds were further accurately determined by the HPLC-ESI-MS/MS method (Li et al., 2014), including caffeoylquinic acids (3-O-caffeoyl quinic acid, 4-O-caffeoyl quinic acid and chlorogenic acid), dicaffeoyl derivatives (rosmarinic acid and rosmarinic acid-4-O-β-D-glucoside), and coumarins (isofraxidin and eleutheroside B1). Among them, eleutheroside B_1_, isofraxidin and rosmarinic acid-4-O-β-D-glucoside are rare natural products (Li et al., 2014; Liu et al., 2017). An integral analysis of absorbed constituents and their metabolites of *S. glabra* reveals that chlorogenic acid (C), isofraxidin (I) and rosmarinic acid (R) all exist as the parent absorbed forms in rat plasma (Xiong et al., 2016), although these polyphenol components may have low oral bioavailability (Teng and Chen, 2018). It is known that using the entire plant or the mixture of major bioactive components to treat diseases is one of the typical characteristics of Chinese medicine. However, the potential synergistic effects existing in the major bioactive compounds are unknown. This study aims to investigate whether C + R + I have protective effects in suppressing LPS-induced ALI and attempts to identify the mechanisms in the NF-κB and MAPK signaling pathways. The potential combined anti-inflammatory effect mechanisms between them have been studied and discussed.

## Materials and Methods

### Materials

Chlorogenic acid and rosmarinic acid were purchased from Chengdu Biopurify Phytochemicals Ltd. (Chengdu, China), isofraxidin was purchased from the National Institute for the Control of Pharmaceutical and Biological Products (Beijing, China), and their purities were more than 98%. RAW 264.7 macrophages cell line was purchased from American Type Culture Collection (Rockville, MD, United States). Antibodies, including anti-NF-κB, anti-IκB, anti-INOS, anti-COX-2, anti-P38, anti-JNK, anti-ERK and anti-GAPDH were purchased from Cell Signaling Technologies (Pickering, ON, CAN). Lipopolysaccharide (LPS) was purchased from Sigma (Sigma, MO, United States). Fetal bovine serum, Dulbecco's Modified Eagle's Medium and penicillin/streptomycin solution were purchased from HyClone (Logan, UT, United States). Cytometric Bead Array (CBA) Kits were purchased from eBioscience (San Diego, CA, United States). Enhanced Chemiluminescence (ECL) Solution Kit and secondary antibodies was purchased from Millipore (Billerica, MA, United States).

### Animals

Adult female and male BALB/c mice (8–10 weeks old, 18–22 g weight) were purchased from Guangdong Laboratory Animal Medicine Center. Mice were allowed to acclimatize to the laboratory for at least 7 days in a standard laboratory with controlled temperature (22 ± 2°C), relative humidity (45–55%), artificial light with a 12 h light and 12 h darkness cycle, and provided free access to food and water under a specific pathogen-free environment. Animal Experimental Ethics Committee of The Second Affiliated Hospital of Guangzhou University of Chinese Medicine approved the protocols.

### Mice Model of Lipopolysaccharide-Induced Acute Lung Injury

The procedures for induction of ALI by LPS in mice were performed as previously described in previous reports (Niu et al., 2012). Briefly, mice were randomly divided into six groups, including control, model, dexamethasone (DEX), and three experimental groups C + R + I high dose (50, 20, and 20 mg/kg), C + R + I middle dose (25, 10, and 10 mg/kg), C + R + I low dose (12.5, 5, and 5 mg/kg). The experimental groups were intraperitoneally (i.p.) injected with 50, 25, 12.5 mg/kg of chlorogenic acid, 20, 10, and 5 mg/kg of rosmarinic acid, and 20, 10, and 5 mg/kg of isofraxidin once a day, respectively. The administration dose for *in vivo* investigation was chosen according to previous studies (Jiang et al., 2009; Niu et al., 2012; Meng et al., 2014). In contrast, the groups of control, model, and dexamethasone (DEX) received an i. p. injection of saline solution. Three days later, the mice fasted for about 12 h and then received 50 μL LPS solution (10 μg LPS) through intratracheal (i.t.) instillation. Each group was dosed once again after the first administration for 1 h, whereas the positive control group was injected with 2 mg/kg DEX.

Upon LPS stimulation for 24 h, the mice were sacrificed with 50 mg/kg pentobarbital. Blood and lung samples were collected from ten animals, individually. The blood was then used for iNOS and TNF-α analysis, the lung samples were used for MPO, SOD, IL-1β and COX-2 determination, histopathological and immunohistochemistry studies. Bronchopleural lavage fluid was collected from the other six mice for leukocytes analysis.

### Molecular Docking

The targets for chlorogenic acid, rosmarinic acid, and isofraxidin were predicted by molecular docking. The crystal structures of ligand-protein complexes were downloaded from the RSCB Protein Data Bank. The PDB ID for SOD, MPO, HO-1, ERK, iNOS, COX-2, JNK, NF-kB, and p38 MAPK were 5O3Y (Wright et al., 2013), 4C1M (Forbes et al., 2013), 6EHA (Mucha et al., 2019), 4QTB (Chaikuad et al., 2014), 3E7G (Garcin et al., 2008), 5IKR (Orlando and Malkowski, 2016), 4QTD (Chaikuad et al., 2014), 6NV2 (Wolter et al., 2020) and, 3ZS5 (Azevedo et al., 2012), respectively. The binding site for each protein was defined by the occupied space of the corresponding ligand in the crystal structure. The calculations were performed by Autodock 4.2.6 [22,89] with default parameters. The binding energy was used to evaluate the binding affinity between each compound and each protein.

### Cell Culture and Treatments

RAW 264.7 macrophages were maintained in DMEM supplemented with 10% heat-inactivated FBS, 10,000 U/ml penicillin and 10,000 μg/ml streptomycin at 37°C in an incubator with a humidified atmosphere and 5% CO_2_. RAW 264.7 macrophages were cultured in the presence of LPS (100 ng/ml) with C + R + I (0.03, 0.06, and 0.06 mmol). The choice of concentration for C + R + I was also according to reference (Huang et al., 2009; Shan et al., 2009; Niu et al., 2012) and CCK8 pre-experiment.

### Evaluation of TNF-α, COX-2, iNOS, and IL-6 Inflammatory Cytokines

The levels of inflammatory cytokines, including TNF-α, COX-2, iNOS, and IL-6 of the lung in LPS-induced pulmonary injury were measured using a CBA assay kit. Then, these concentrations were calculated via the standard curves of TNF-α, iNOS, COX-2, and IL-6.

### Determination the Production of Nitric Oxide

To analyze the production of nitric oxide, RAW264.7 cells in 6-well plates were cultured for 3 h with or without C + R + I and then incubated with LPS for 24 h. NO production was then evaluated by quantifying the nitrite in the culture medium using the Griess reagent. Briefly, 100 μL of the collected supernatant was mixed with Griess reagent and incubated at room temperature for 15 min. Absorbance values at 540 nm were recorded, and the levels of nitric oxide were determined according to the standard curve of sodium nitrite ranging from 0.1 to 10 μM.

### Western Blot Analysis

After treatments, RAW264.7 cells were washed with pre-cooling PBS and lyzed with RIPA (containing proteinase inhibitor and phospho-protein inhibitor), then centrifuged at 12,000 rpm for 10 min at 4°C and the lyzed supernatant was collected. Protein content of the lyzed cells was analyzed by BCA assay kit. Protein samples were loaded, separated by sodium dodecyl and transferred to 0.45 µm polyvinylidene difluoride (PVDF) membranes. 0.5% BSA in 1× Tris-buffered saline (TBS) containing 0.05% Tween-20 was used to block membranes by 1 h incubation. Protein samples were then separated by sulfate–polyacrylamide gel electrophoresis (SDS–PAGE) on 10% gels. Membranes were treated with primary antibodies at 4°C overnight and then treated with horseradish peroxidase-coupled secondary antibody for 2 h at 4°C. Finally, the membranes were washed with TBST after each antibody-binding reaction. Enhanced chemiluminescence (ECL) kit was used to detect the protein.

### Histopathological Study

The lung tissues were fixed in 4% paraformaldehyde and then embedded in paraffin for sectioning. Several sections were stained with hematoxylin and eosin to examine the histological differences.

### Immunofluorescence

The lung samples were fixed with 4% paraformaldehyde, followed by washes with PBS. Then tissues were permeabilized with 0.1% Triton-X 100 for 10 min, blocked with 5% donkey serum for 1 h and incubated with P-p65, P-p38, and COX-2 antibody separately overnight at 4°C. Following incubation with goat serum (secondary antibody) for 1 h at room temperature, the samples were then incubated with hematoxylin stain.

### Statistical Analysis

Statistical analysis was performed using Student t test, or one-way ANOVA followed by Tukey’s post-test. The results are expressed as mean ± standard error of the mean (s.e.m.) and significance was set as *p* < 0.05.

## Results

### Effects of C + R + I on Lung Histopathology in Lipopolysaccharide-Induced Acute Lung Injury

**FIGURE 1:**
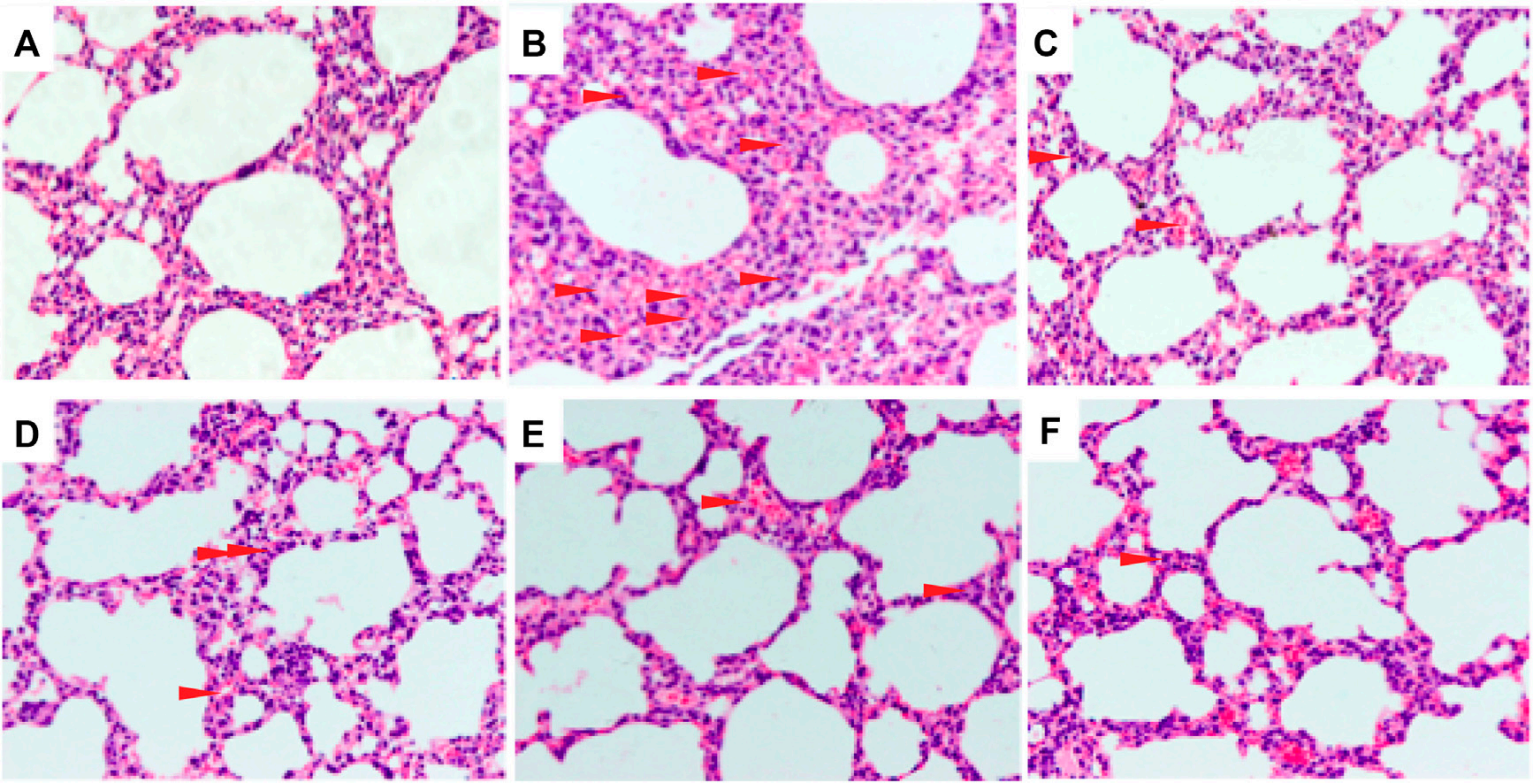
Effects of C + R + I pretreatment on pathological changes in lung tissues of LPS-induced ALI (original magnification ×200); **(A)** Control; **(B)** LPS; **(C)** 1 mg/kg dexamethasone + LPS; **(D)** C + R + I low dose + LPS; **(E)** C + R + I middle dose + LPS, and; **(F)** C + R + I high dose + LPS. Data were expressed as means ± SEM of three independent experiments. *: *p* < 0.05, **p < 0.01 as compared to the LPS group without C + R + I, #*p* < 0.05, ##*p* < 0.01 as compared to the normal control group without C + R + I. Effects of C + R + I on the inflammatory cell infiltration in LPS-induced ALI.

To evaluate the effect of C + R + I on ALI, we examined the degree of damage that occurred in the lung of LPS-administered mice pretreated with or without C + R + I by H&E staining. No histopathological changes were observed from the control group under the optical microscope (Figure 1A), while changes in lung structure, pulmonary edema, and the accumulation of inflammatory cells were observed in animals without C + R + I pretreatment (Figure 1B). As expected, 1 h of C + R + I pretreatment reduced the LPS-induced lung damage at all low, middle and high doses (Figure 1D–F–F), similar results were observed in mice pretreated with DEX (Figure 1C). To sum up, these results suggest that C + R + I can improve the condition of ALI induced by LPS.

**FIGURE 2:**
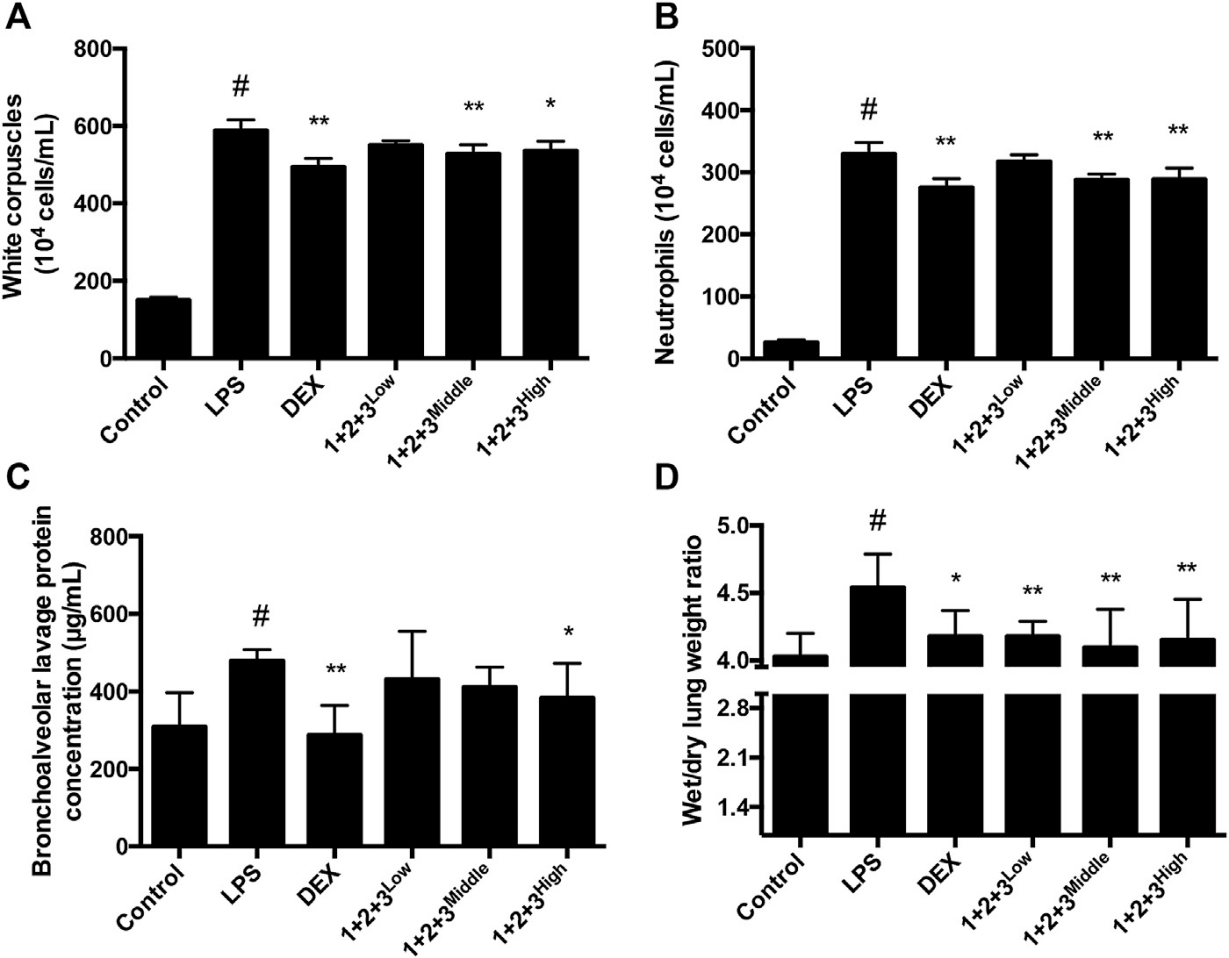
Effects of C + R + I (1 + 2+3) on the infiltration of inflammatory cells in LPS-induced ALI. PBS or C + R + I was intraperitoneally injected 30 min before intratracheal administration of LPS (100 μg/50 μl saline) or saline in mice ;**(A)** White corpuscles; **(B)** neutrophils; **(C)** bronchoalveolar lavage protein; **(D)** wet/dry lung weight ratio. *p < 0.05, **p < 0.01 as compared to the LPS group without C + R + I, #*p* < 0.05, ##*p* < 0.01 as compared to the normal control group without C + R + I. Effects of C + R + I on SOD and MPO production in LPS-induced ALI.

ALI is characterized by the infiltration of polynuclear neutrophil, white corpuscles and bronchialveolar. To investigate whether C + R + I could reduce the inflammatory cells infiltration *in vivo*, we detected the changes in inflammatory cells infiltration occurring in the lung of LPS-induced mice pretreated with or without C + R + I. As showed in Figure 2A-C, significant reductions in the inflammatory cell infiltration were observed in the BALF with C + R + I pretreatment. The wet/dry lung weight ratio was also obviously decreased compared with the LPS group (Figure 2D). Results demonstrated that C + R + I at the middle dose had protective effect on ALI mice by decreasing inflammatory cells infiltration.

**FIGURE 3:**
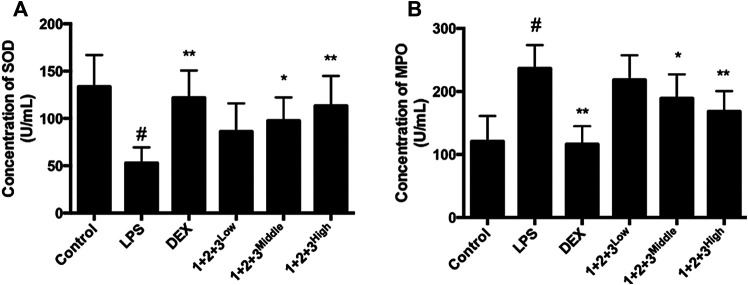
Effects of C + R + I (1 + 2+3) on the pro-inflammatory cytokine secretion in serum in LPS-induced ALI; **(A)** Concentration of SOD; **(B)** concentration of MPO. Values are expressed as the mean 7 SD (n1⁄43 or 4 in each group). *p < 0.05, **p < 0.01 as compared to the LPS group without C + R + I, #*p* < 0.05, ##*p* < 0.01 as compared to the normal control group without C + R + I. Effects of C + R + I on TNF-α and IL-1β, COX-2 and iNOS production in LPS-induced ALI.

Oxidative stress has a critical role in the pathophysiology of ALI. The anti-oxidative enzyme SOD is consumed during amelioration of ALI. In this study, we found that SOD secretion was significantly inhibited in LPS-induced mice, while the pretreatment with C + R + I recovered the LPS-induced reduction in a concentration-dependent manner (Figure 3A).

We further measured the MPO influence of C + R + I treatment, which is the marker for macrophage infiltration. We found that MPO secretion was increased in the lung tissue of LPS-induced mice. As expected, the up-regulation of MPO was prevented in mice pretreated with C + R + I (Figure 3B). The results showed that C + R + I could protect LPS-induced ALI mice by reducing macrophage activation.

**FIGURE 4:**
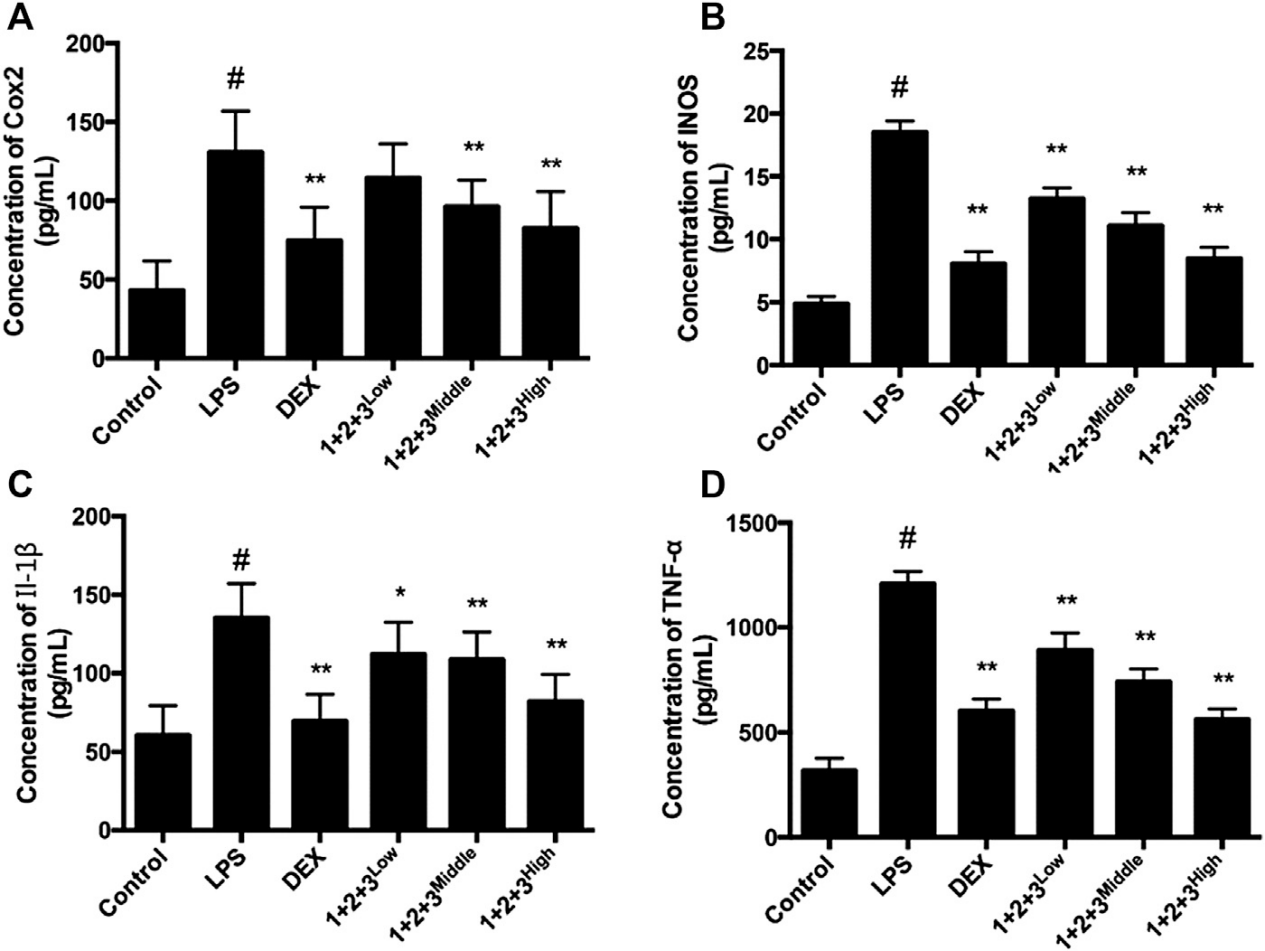
Effects of C + R + I (1 + 2+3) on the pro-inflammatory cytokine secretion in serum in LPS-induced ALI. Concentration of; **(A)** COX-2; **(B)** iNOS; **(C)** IL-1β, and,; **(D)** TNF-α. Values are expressed as the mean 7 SD (n1⁄43 or 4 in each group). *p < 0.05, **p < 0.01 as compared to the LPS group without C + R + I, #*p* < 0.05, ##*p* < 0.01 as compared to the normal control group without C + R + I. Effects of C + R + I on inhibition of MAPK-NF-κB in LPS-induced ALI.

Pro-inflammatory cytokines, including TNF-α, iNOS, COX-2 and IL-6 are major mediators involved in recruitment of macrophages into the lungs during LPS-induced pulmonary injury. In this study, the TNF-α, iNOS, COX-2,, and IL-6 levels in the serum of ALI were tested via Liquid chip method. LPS induced TNF-α, iNOS, COX-2 and IL-6 production by the macrophages in ALI, while the productions were decreased by C + R + I in a dose-dependent manner (*p* < 0.05 or 0.01, Figure 4A–D–D). The results demonstrated that C + R + I decreased the expression of pro-inflammatory cytokines.

In other tissue contexts, cooperativity of p65-NF-κB, *p*-JNK and iNOS has been shown to reflect cross talk on signaling pathways, particularly NF-κB signaling. To detect.

**FIGURE 5:**
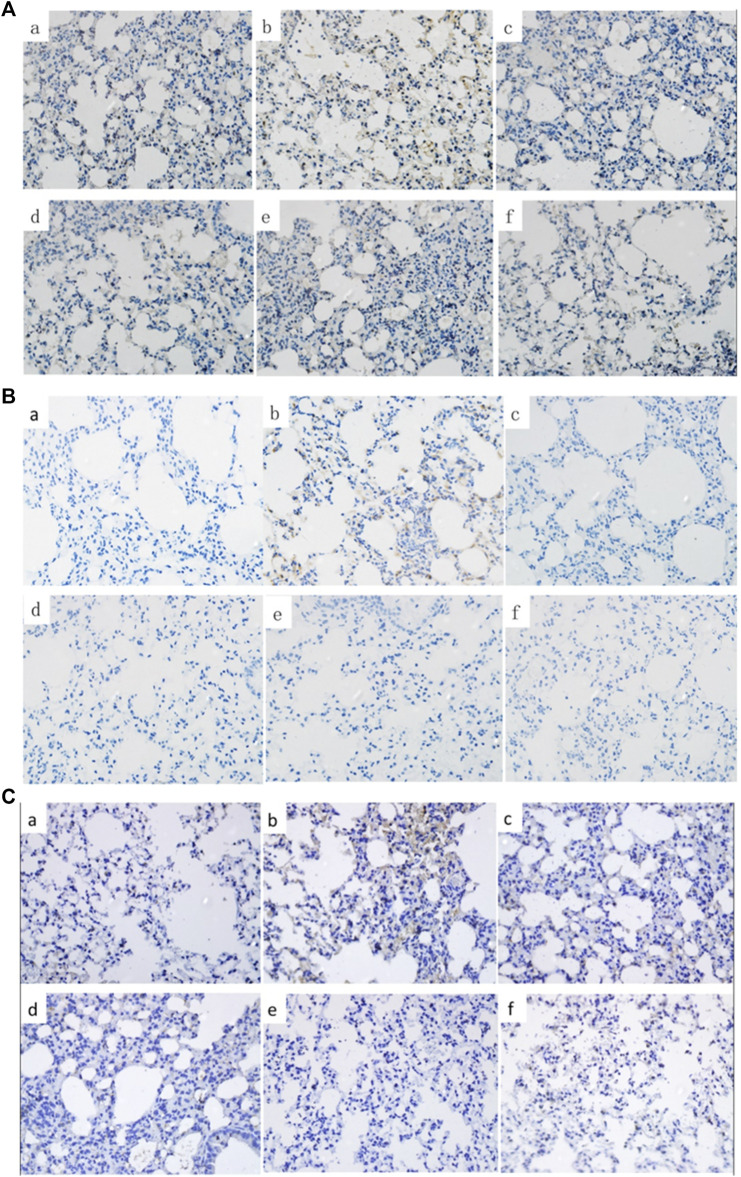
Effects of C + R + I on the protein of P-p65, P-p38 and COX-2 in LPS-induced ALI. Immunostaining of; **(A)** activated P-p65; **(B)** activated P-p38; **(C)** COX-2. Effects of C + R + I on NO, HO-1, SOD and MPO in LPS-induced ALI.

Whether MAPK-NF-κB signaling pathway was involved, the immune cytochemical analyses were used to measure protein expression of the lung in LPS-induced ALI with or without C + R + I pretreatment. LPS-induced mice resulted in phosphorylation of JNK, ERK and p38 MAPK in lung tissue. Herein, we found that pretreatment with C + R + I decreased the phosphorylation of NF-κB p65, and p38 in the lung of LPS-induced ALI. (Figure 5A–C–C).

**FIGURE 6:**
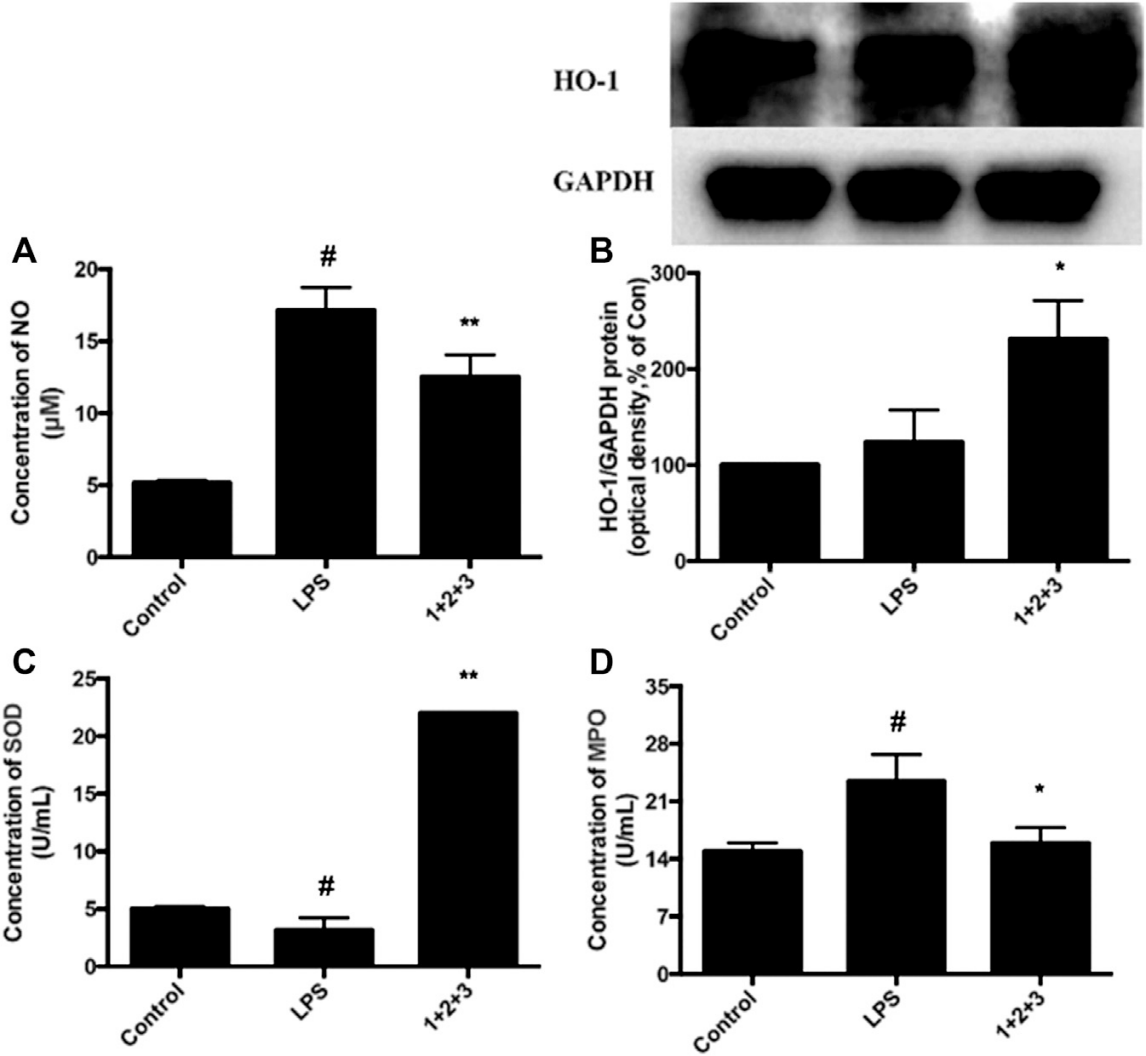
Effects of C + R + I (1 + 2+3) on LPS-induced antioxidative enzyme activation in RAW264.7 cells; **(A)** NO **(B)** HO-1, and; **(C)** SOD, and; **(D)** MPO. Values are indicated as the mean of four mice per group. *p < 0.05, **p < 0.01 as compared to the LPS group without C + R + I, #*p* < 0.05, ##*p* < 0.01 as compared to the normal control group without C + R + I. The effect of C, R, and I on the inhibition of MAPK-NF-κB signaling pathway.

The anti-oxidative protein HO-1 is expressed during the relieving of ALI. The effect of C + R + I on HO-1 expression in LPS-stimulated RAW 264.7 cells were detected by western-blot. As shown in Figure 6B, C + R + I increased LPS-stimulated HO-1 expression, suggesting that C + R + I can decrease LPS-stimulated oxidative stress (*p* < 0.05). The ant-oxidative enzyme SOD is consumed during amelioration of ALI. In this study, we found that SOD is significantly inhibited in LPS-induced mice compared to the control group, while the pretreatment with C + R + I increased the anti-oxidative enzymes’ activity (Figure 6C). MPO activity is a marker for macrophage and neutrophil infiltration and activation. We measured the ability of C + R + I in influence of MPO activity and found that MPO was significantly downregulated in lung tissue of ALI (Figure 6D). In addition, it is well known that NO are important active molecules with key roles in macrophage. In this study, we found that pre-treatment with C + R + I also significantly reduced the production of NO in lung tissue of ALI (*p* < 0.05) (Figure 6A). These results demonstrated that C + R + I had a protective effect on LPS-induced ALI mice by inhibiting macrophage infiltration and activation.

**FIGURE 7:**
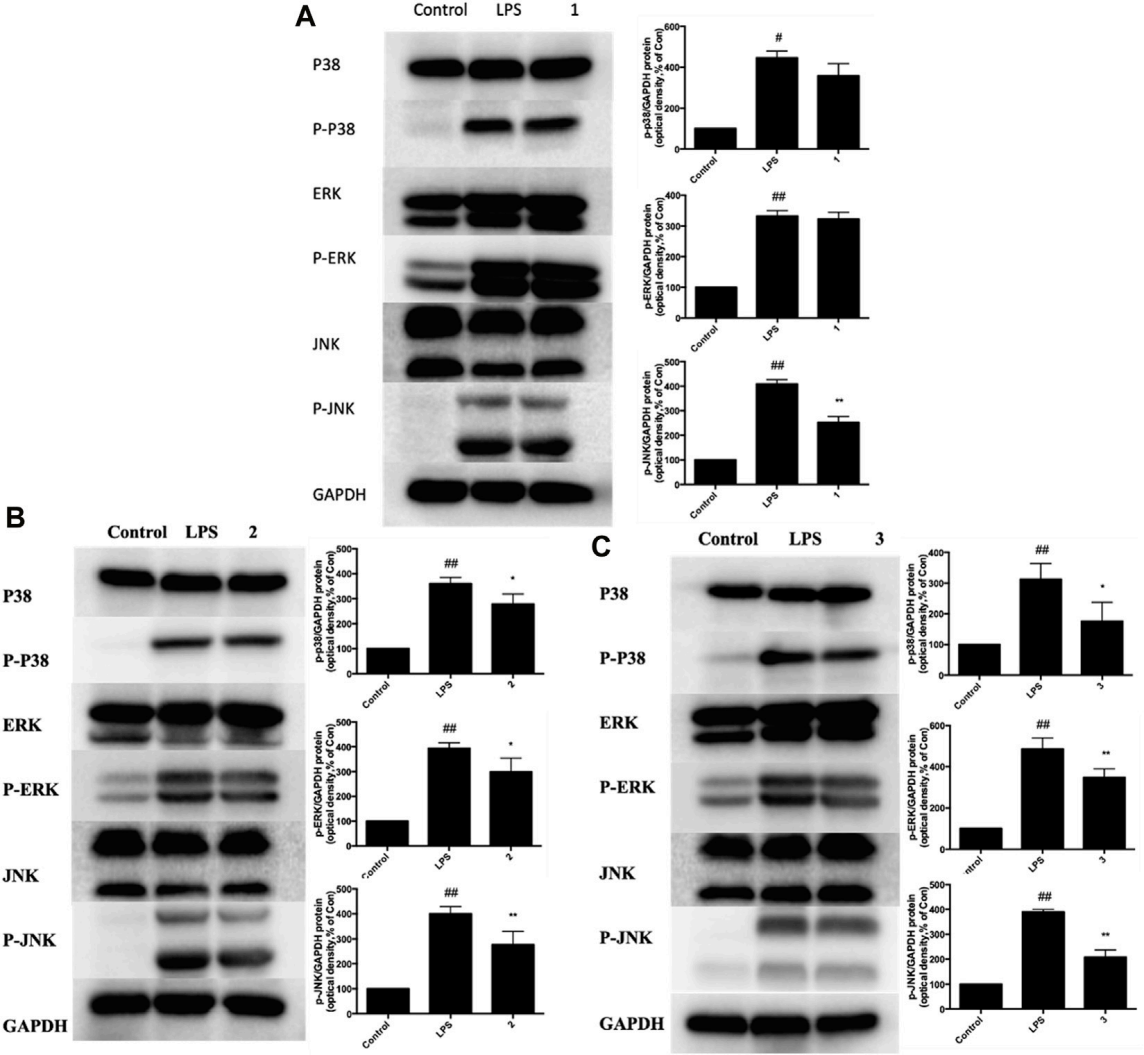
Effects of C, R, I (1, 2, 3) on p38 MAPK, ERK, and JNK activation in RAW264.7 cells stimulated by LPS. Pretreatment with; **(A)** C; **(B)** R; **(C)** I; Values are indicated as the mean of four mice per group. *p < 0.05, **p < 0.01 as compared to the LPS group, #*p* < 0.05, ##*p* < 0.01 as compared to the normal control group. Combined effect of C + R + I on inhibiting the activation of MAPK-NF-κB signaling pathway.

Next, we investigated whether C, R, and I used alone could modulate the expression of key molecules in MAPK-NF-κB signaling pathway in RAW264.7 cells stimulated by LPS. Briefly, cells were pretreated with C, R or I alone for 3 h, following exposure to LPS for 24 h. Western blot analysis was then used to measure the protein expression of p38-MAPK, P-JNK, and P-ERK. After treatment with LPS, the protein expression of MAPK-NF-κB pathway, including P-p38, P-JNK, and P-ERK significantly increased. As shown in Figure 7A, pretreatment with C alone reduced the expression of P-JNK protein by 38%, but not obviously influenced the expression of P-p38 and P-ERK protein. In addition, the expression of P-p38, P-JNK and P-ERK protein was inhibited but not significantly influenced by R and I alone (Figure 7B, C).

**FIGURE 8:**
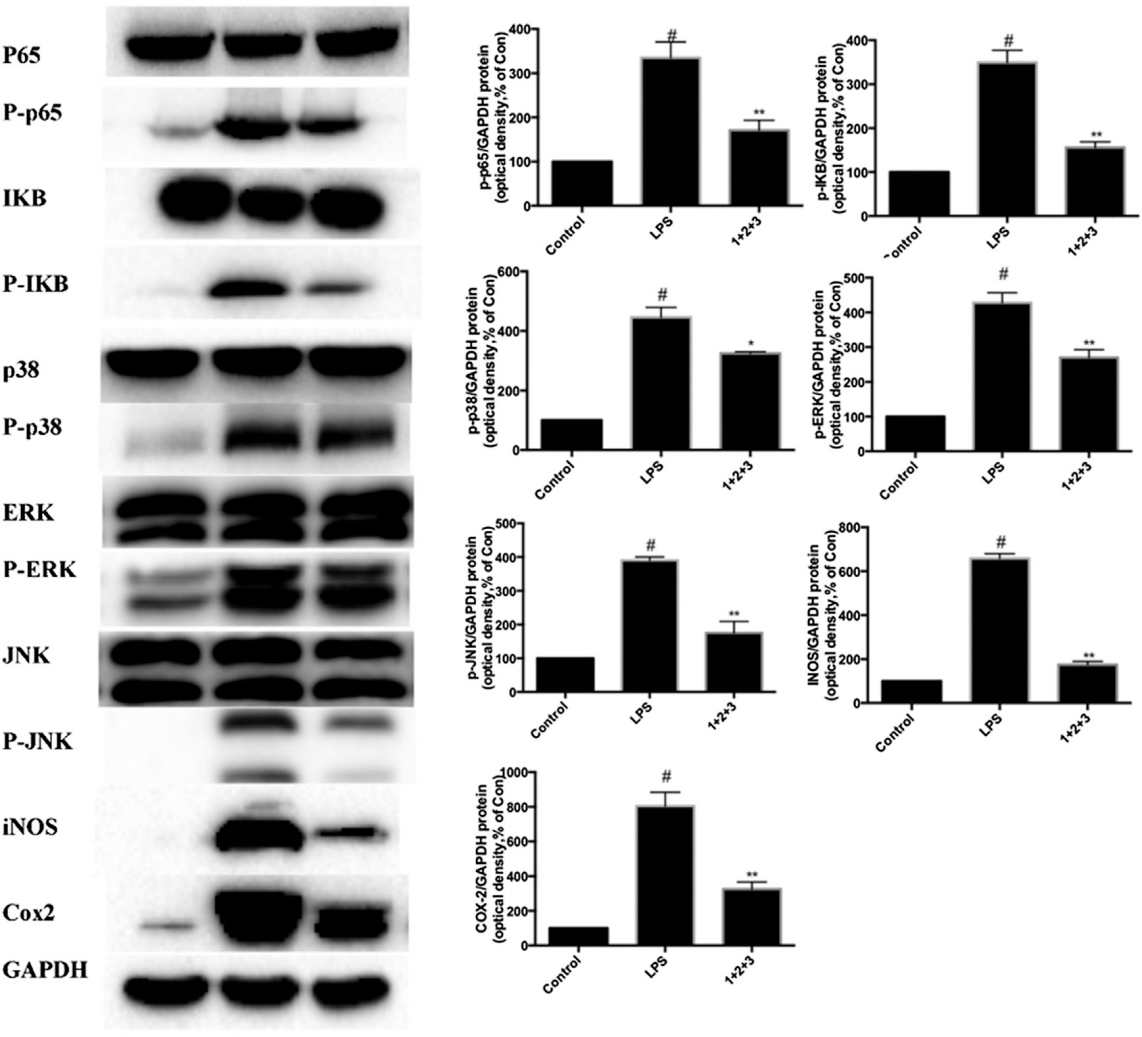
Combined effects of C + R + I (1 + 2+3) on suppressing the activation of MAPK-NF-κB signaling pathway in RAW264.7 cells stimulated by LPS. Lungs were harvested and analyzed by Western blotting. Values are indicated as the mean of four mice per group. *p < 0.05, **p < 0.01 as compared to the LPS group without C + R + I, #*p* < 0.05, ##*p* < 0.01 as compared to the normal control group without C + R + I. C, R, and I have multiple targets.

We further investigated whether C + R + I could synergistically increase and modulate the expression of key molecules in MAPK-NF-κB signaling pathway. Combined pretreatment with C + R + I, with the same concentration used alone for 3 h following exposure to LPS for 24 h significantly decreased the expressions of IκB, COX-2, iNOS, NF-κB P-p65, P-p38, P-JNK, and P-ERK (*p* < 0.05 or *p* < 0.01) (Figure 8). Through comprehensive analysis of the data, an obvious synergistic effect was found for combination use of C + R + I.

**FIGURE 9:**
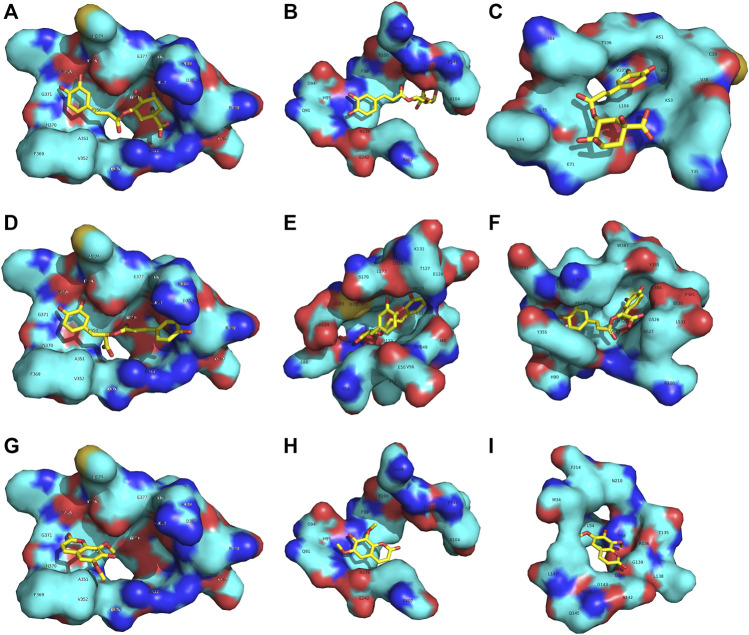
C, R and I have multiple targets **(A–C)** C had a good affinity with the inhibitor binding site of iNOS and MPO **(D–E)** R can target iNOS, ERK and COX-2 **(G–I)** I can interact with iNOS, MPO and HO-1.

The binding affinity between each compound and nine proteins in these pathways were calculated by molecular docking. The results demonstrated that the three compounds can interact with multiple targets, and similar phenomena have been reported (Teng et al., 2016; Chen et al., 2018; Chen et al., 2019a; Chen et al., 2019b). C had a good affinity with the inhibitor binding site of iNOS (the binding energy was -8.39 kcal/mol), MPO (-7.34 kcal/mol) and p38 MAPK (-6.94 kcal/mol) (Figure 9A–C). R can target iNOS (-7.28 kcal/mol), ERK (-7.11 kcal/mol) and COX-2 (-6.96 kcal/mol) (Figure 9D–E). I can interact with iNOS (-6.28 kcal/mol), MPO (-7.38 kcal/mol) and HO-1 (-6.76 kcal/mol) (Figure 9G–I). It is interesting that the three compounds had a common target (iNOS), which indicated an inhibitory effect on this pro-inflammatory cytokine. Therefore, the combined effect of C + R + I on suppressing MAPK-NF-κB signaling pathway would attribute to the interactions through multiple targets.

## Discussion

COVID-19 infections may induce diffuse alveolar damage and overproduction of inflammatory factors and ALI/ARDS is the central complication in the patients with COVID-19 infection (Chen et al., 2016). LPS-induced ALI in mice is a widely used animal model to examine ALI *in vivo*; this method can mimic the pathological characteristics of human ALI (Matthay et al., 2019; Zhou et al., 2019). In this study, LPS was delivered to mice through intratracheal instillation to develop ALI. Pulmonary cells and alveolar macrophages of ALI release cytotoxic and inflammatory mediators, such as NO, and secrete pro-inflammatory cytokines, including iNOS, tumor necrosis factor TNF-α, interleukin (IL)-6 and IL-1β. The pro-inflammatory cytokines TNF-α and IL-1β stimulate the neighboring cells to generate more effective pro-inflammatory cytokines such as IL-6, and subsequently mediate the recruitment of PMNs, macrophages, and lymphocytes. *S. glabra* could reduce the generation of IL-6 and TNF-α in LPS-treated RAW264.7 cells (Tsai et al., 2017; Yang et al., 2019), and in acute lung damage in LPS-intoxicated rats (Tsai et al., 2017). In this paper, we studied the combined effect of three main constituents in *S. glabra* (chlorogenic acid, rosmarinic acid and isofraxidin) in treating and preventing ARDS. Our data showed that C + R + I greatly decreased the secretion of TNF-α, IL-1β, and IL-6 in serum and leukocyte in the lung of LPS-induced ALI, which suggested that C + R + I might reduce inflammatory cells infiltration into lung by reducing the production of pro-inflammatory cytokines (Liu, 2016).

NF-κB pathways is key for regulating inflammatory response by decreasing the inflammatory cells infiltration in ALI (Arcambal et al., 2019). Phosphorylation of p65 plays central role in the NF-κB signaling cascade, and IκB is associated with p65. Our study found that C + R + I decreased the levels of LPS-induced phosphorylated NF-κB protein expression. To further evaluate whether IκB participated in this process, we detected the cytoplasmic levels of IκB protein. Our data demonstrated that C + R + I can suppress the expression and degradation of IκB in LPS-stimulated RAW264.7 cells.

Heat shock protein (HO-1) has indispensable effects on restraining the NF-κB pathway. HO-1 is an enzyme catalyzing substrate to carbon monoxide and biliverdin, which participates in anti-oxidant and anti-inflammatory processes (Kim et al., 2016). Our results indicated high expression of HO-1 in mice treated with LPS group. This data revealed that LPS activates NF-κB in macrophages, which could be effectively depressed by C + R + I. Meanwhile, we found that pretreatment with C + R + I inhibited the expression of INOS and COX-2 in LPS-induced RAW 264.7 cells.

Furthermore, MAPK is one of the upstream kinases in the NF-κB p65 phosphorylation pathway, in which MAPK activation is necessary at threonine and tyrosine residues within the kinase's activation loop. Phosphorylation of p38 MAPK and JNK induced by LPS can be inhibited by *S. glabra* (Li et al., 2019). Our study demonstrated that LPS stimulation resulted in phosphorylation of ERK, p38 MAPK, and JNK in lung tissue, while C + R + I pretreatment prevented these manifestations. Parallel trends found that C + R + I inhibit the phosphorylation of MAPK and NF-κB p65. Western-blot results showed that C + R + I could decrease the expression of *p*-ERK, P-p38 and *p*-JNK. In addition, MAPK pathway has an important role in macrophage activation and infiltration (Shu et al., 2018). Superoxide dismutase (SOD) is an antioxidant metal enzyme in organisms and is related to the activation of P38 signaling pathway. Our study showed that pretreated with C + R + I was able to promote the activation of SOD.

MPO is a heme-containing peroxidase abundantly expressed in macrophage (Li et al., 2015; Wu et al., 2019). It catalyzes the generation of hypochlorous acid by consuming hydrogen peroxide, which in turn decreases the secretion of TNF-α, IL-6, and IL-1β (Fernandez and Walters, 1996; Wu et al., 2019). In this study, we found that C + R + I could alleviate LPS-induced acute lung injury of mice. It decreased LPS-induced ALI effectively by inhibiting infiltration of leukocytes and histopathological changes in the lungs. MPO was obviously decreased after the treatment with C + R + I, which induced over-production of NO from iNOS that is important mediator in the pathogenesis of inflammation. Results showed that IL-1β, IL-6, and TNF-αsuppressed downstream signaling pathways, thus decreasing iNOS and COX-2 protein expression. These results indicated that the main components (chlorogenic acid, isofraxidin and rosmarinic acid) from *Sarcandra glabra* exerted anti-inflammatory effect through multiple targets.

**FIGURE 10:**
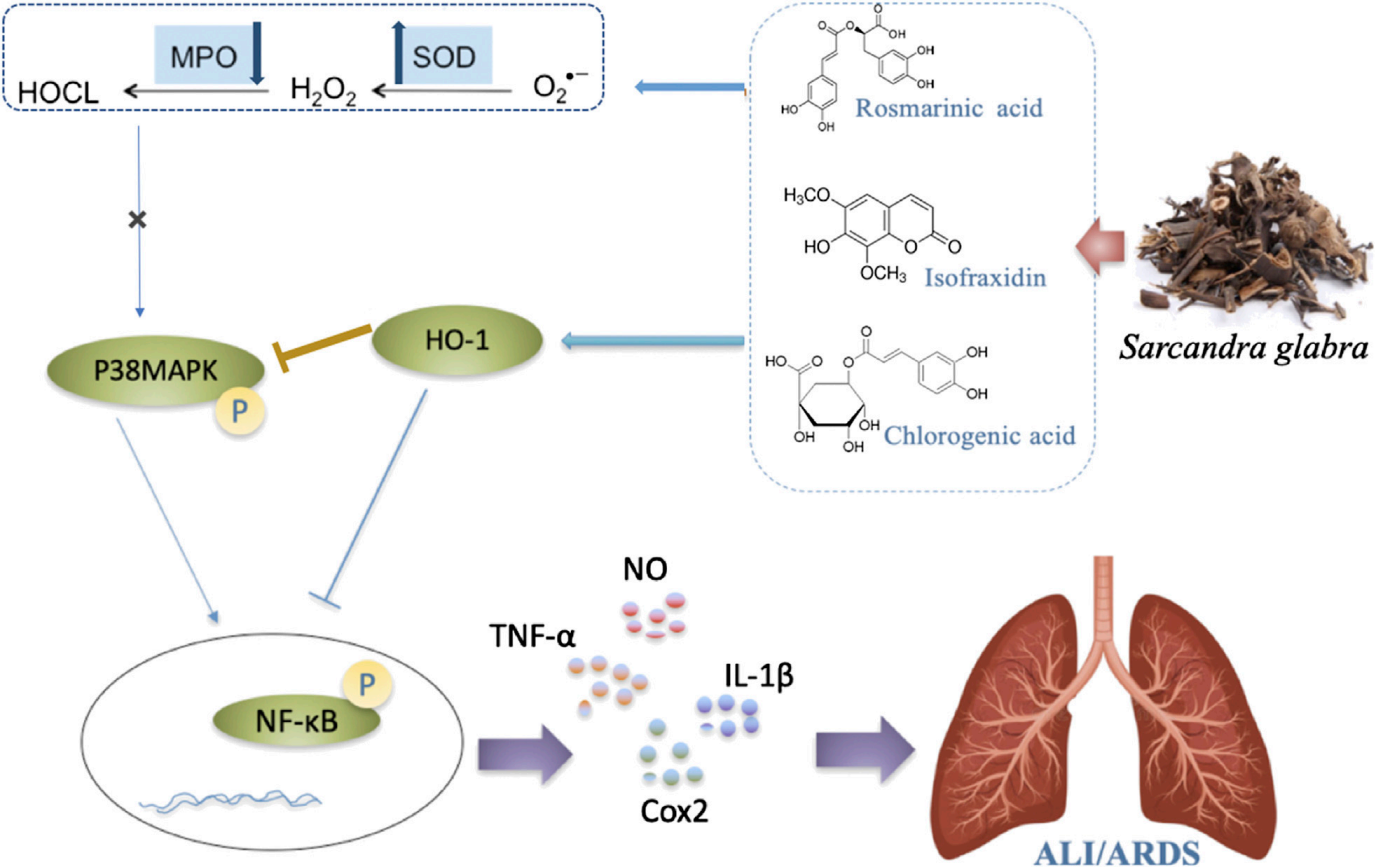
Possible intervening molecular mechanisms of C + R + I from Sarcandra glabra on LPS-induced ALI.

In summary, the action mechanism of the combination of three main constituents C + R + I from *S. glabra* were studied. The result ravels that C + R + I target MAPK-NF-κB pathway in LPS stimulated RAW 264.7 cells, as well as in mice with LPS-induced acute lung injury. C + R + I extracted from *S. glabra* decreased oxidative stress and inflammatory response by inhibiting the activation of the macrophages resulting in decreasing the secretion of NO, IL-6, and TNF-α (Figure 10). In conclusion, this study provides new views into the potential treatment with *S. glabra* and shows a promising candidate for treating ALI/ARDS caused by pneumonia, COVID-19, appendicitis, and so on.

## Data Availability Statement

The original contributions presented in the study are included in the article/Supplementary Material, further inquiries can be directed to the corresponding authors.

## Ethics Statement

The animal study was reviewed and approved by Ethics Committee of Experimental Animals of Guangdong Hospital of Traditional Chinese Medicine.

## Author Contributions

C-PL and J-HL mainly for the experiment carrying out and article writing, XL mainly for experiment design, J-XL data sorting and statistics, JG for molecular docking, FL, BY, JL, and the QW mainly to modify articles; XZ, L-ML, H-LY, and LW focuses on the language of the article polish.

## Funding

This work was financially supported by the Guangdong Basic and Applied Basic Research Foundation (No. 2019A1515111108), the National Natural Science Foundation of China (Nos. 81202888, 81573708 & No. 81803919), Guangdong Natural Science Funds for Distinguished Young Scholar (No. 2015A030306049). The funding sources had no involvement in study design; in the collection, analysis and interpretation of data; in the writing of the report; and in the decision to submit the article for publication.

## Conflict of Interest

The authors declare that the research was conducted in the absence of any commercial or financial relationships that could be construed as a potential conflict of interest.

## References

[B1] Arcambal A. Taïlé J. Rondeau P. Viranaïcken W. Meilhac O. Gonthier P. M. (2019). Hyperglycemia modulates redox, inflammatory and vasoactive markers through specific signaling pathways in cerebral endothelial cells: insights on insulin protective action. Free Radic. Biol. Med. 130, 59–70. 10.1016/j.freeradbiomed.2018.10.430 30359759

[B2] Azevedo R. van Zeeland M. Raaijmakers H. Kazemier B. de Vlieg J. Oubrie A. (2012). X-ray structure of p38alpha bound to TAK-715: comparison with three classic inhibitors. Acta Crystallogr. D Biol. Crystallogr. 68, 1041–1050. 10.1107/S090744491201997X 22868770

[B3] Chaikuad A. Tacconi E. M. Zimmer J. Liang Y. Gray N. S. Tarsounas M. (2014). A unique inhibitor binding site in ERK1/2 is associated with slow binding kinetics. Nat. Chem. Biol. 10, 853–860. 10.1038/nchembio.1629 25195011 PMC4687050

[B4] Chen B. Yang Z. Yang C. Qin W. Gu J. Hu C. (2018). A self-organized actomyosin drives multiple intercellular junction disruption and directly promotes neutrophil recruitment in lipopolysaccharide-induced acute lung injury. FASEB J, fj201701506RR. 10.1096/fj.201701506RR 29879372

[B5] Chen L. Lin X. Xiao J. Tian Y. Zheng B. Teng H. (2019b). Sonchus oleraceus Linn protects against LPS-induced sepsis and inhibits inflammatory responses in RAW264.7 cells. J. Ethnopharmacol. 236, 63–69. 10.1016/j.jep.2019.02.039 30802614

[B6] Chen L. Teng H. Cao H. (2019a). Chlorogenic acid and caffeic acid from Sonchus oleraceus Linn synergistically attenuate insulin resistance and modulate glucose uptake in HepG2 cells. Food Chem. Toxicol. 127, 182–187. 10.1016/j.fct.2019.03.038 30914352

[B7] Chen L. Teng H. Fang T. Xiao J. (2016). Agrimonolide from Agrimonia pilosa suppresses inflammatory responses through down-regulation of COX-2/iNOS and inactivation of NF-kappaB in lipopolysaccharide-stimulated macrophages. Phytomedicine 23, 846–855. 10.1016/j.phymed.2016.03.016 27288920

[B8] Chen L. Teng H. Jia Z. Battino M. Miron A. Yu Z. (2018). Intracellular signaling pathways of inflammation modulated by dietary flavonoids: the most recent evidence. Crit. Rev. Food Sci. Nutr. 58, 2908–2924. 10.1080/10408398.2017.1345853 28682647

[B9] de Oliveira M. T. P. de Sá Coutinho D. de Souza É T. Guterres S. S. Pholmann A. R. Silva P. M. R. (2019). Orally delivered resveratrol-loaded lipid-core nanocapsules ameliorate LPS-induced acute lung injury via the ERK and PI3K/Akt pathways. Int. J. Nanomed. 14, 5215–5228. 10.2147/IJN.S200666 PMC6636190 31371957

[B10] Fernandez M. C. Walters J. (1996). Transcriptional and post-transcriptional regulation of GM-CSF-induced IL-1 beta gene expression in PMN. J. Leukoc. Biol. 59, 598–603. 10.1089/jir.1996.16.33310.1002/jlb.59.4.598 8613710

[B11] Forbes L. V. Sjogren T. Auchere F. Jenkins D. W. Thong B. Laughton D. (2013). Potent reversible inhibition of myeloperoxidase by aromatic hydroxamates. J. Biol. Chem. 288, 36636–36647. 10.1074/jbc.M113.507756 24194519 PMC3868775

[B12] Garcin E. D. Arvai A. S. Rosenfeld R. J. Kroeger M. D. Crane B. R. Andersson G. (2008). Anchored plasticity opens doors for selective inhibitor design in nitric oxide synthase. Nat. Chem. Biol. 4, 700–707. 10.1038/nchembio.115 18849972 PMC2868503

[B13] Huang N. Hauck C. Yum M. Y. Rizshsky L. Widrlechner M. P. McCoy J.-A. (2009). Rosmarinic acid in Prunella vulgaris ethanol extract inhibits lipopolysaccharide-induced prostaglandin E2 and nitric oxide in RAW 264.7 mouse macrophages. J. Agric. Food Chem. 57 (22), 10579. 10.1021/jf9023728 19919113 PMC2795400

[B14] Jiang W. L. Chen X. G. Qu G. W. Yue Z. D. Zhu H. B. Tian J. W. (2009). Rosmarinic acid protects against experimental sepsis by inhibiting proinflammatory factor release and ameliorating hemodynamics. Shock 32 (6), 608. 10.1097/SHK.0b013e3181a48e86 19295475

[B15] Kim K. N. Ko S. C. Ye B. R. Kim M. S. Kim J. Ko E. Y. (2016). 5-Bromo-2-hydroxy-4-methyl-benzaldehyde inhibited LPS-induced production of pro-inflammatory mediators through the inactivation of ERK, p38, and NF-κB pathways in RAW 264.7 macrophages. Chem. Biol. Interact. 258, 108–114. 10.1016/j.cbi.2016.08.022 27569861

[B16] Li K. C. Ho Y. L. Hsieh W. T. Huang S. S. Chang Y. S. Huang G. J. (2015). Apigenin-7-glycoside prevents LPS-induced acute lung injury via downregulation of oxidative enzyme expression and protein activation through inhibition of MAPK phosphorylation. Int. J. Mol. Sci. 16, 1736–1754. 10.3390/ijms16011736 25590301 PMC4307331

[B17] Li L. Huang Q. Wang D. C. Ingbar D. H. Wang X. (2020). Acute lung injury in patients with COVID-19 infection. Clin. Trans. Med. 10, 1–8. 10.1002/ctm2.16 PMC7240840 32508022

[B18] Li X. Zhang Y. Zeng X. Yang L. Deng Y. (2011). Chemical profiling of bioactive constituents in Sarcandra glabra and its preparations using ultra-high-pressure liquid chromatography coupled with LTQ Orbitrap mass spectrometry. Rapid Commun. Mass Spectrom. 25, 2439–2447. 10.1002/rcm.5123 21818803

[B19] Li X. Zhao Y. Huang R. Y. Zhu W. Zeng X. Zhao J. (2014). Simultaneous quantification of 17 bioactive constituents in Sarcandra glabra by liquid chromatography-electrospray ionisation-massspectrometry. Anal Methods 6, 7989–7995. 10.1039/C4AY01259C

[B20] Li Z. Feng H. Wang Y. Shen B. Tian Y. Wu L. (2019). Rosmarinic acid protects mice from lipopolysaccharide/d-galactosamine-induced acute liver injury by inhibiting MAPKs/NF-κB and activating Nrf2/HO-1 signaling pathways. Int. Immunopharm. 67, 465–472. 10.1016/j.intimp.2018.12.052 30597292

[B21] Liang Y. Zeng Z. Gao Y. (2020). Network pharmacology study of prevention mechanism of COVID-19 in Chai Ge Qingyuan Decoction. J. Guizhou Univ. Trad. Chin. Med. 42 (3), 22–25. 10.16588/j.cnki.issn1002-1108.2020.03.006

[B22] Liu J. X. Zhang Y. Hu Q. P. Li J. Q. Liu Y. T. Wu Q. G. (2017). Anti-inflammatory effects of rosmarinic acid-4-O-β-D-glucoside in reducing acute lung injury in mice infected with influenza virus. Antivir. Res. 144, 34–43. 10.1016/j.antiviral.2017.04.010 28461072

[B23] Liu T. Y. (2016). Sarcandra glabra combined with lycopene protect rats from lipopolysaccharide induced acute lung injury via reducing inflammatory response. Biomed. Pharmacother. 84, 34–41. 10.1016/j.biopha.2016.09.009 27631138

[B24] Lv H. Liu Q. Wen Z. Feng H. Deng X. Ci X. (2017). Xanthohumol ameliorates lipopolysaccharide (LPS)-induced acute lung injury via induction of AMPK/GSK3β-Nrf2 signal axis. Redox Biol 12, 311–324. 10.1016/j.redox.2017.03.001 28285192 PMC5345976

[B25] Matthay M. A. Zemans R. L. Zimmerman G. A. (2019). Acute respiratory distress syndrome. Nat. Rev. Dis. Primers. 5 (1), 18. 10.1007/s13312-010-0144-9 10.1007/s13312-010-0144-9 30872586 PMC6709677

[B26] Meng Z. Q. Tang Z. H. Yan Y. X. Guo C. R. Cao L. Huang W. Z. (2014). Study on the anti-gout activity of chlorogenic acid: improvement on hyperuricemia and gouty inflammation. Am. J. Chin. Med. 42 (06), 1450092. 10.1142/S0192415X1450092X 25384446

[B27] Mucha O. Podkalicka P. Mikulski M. Barwacz S. Andrysiak K. Biela A. (2019). Development and characterization of a new inhibitor of heme oxygenase activity for cancer treatment. Arch. Biochem. Biophys. 671, 130–142. 10.1016/j.abb.2019.07.002 31276659

[B28] National Commission of Chinese Pharmacopoeia (2015). The Pharmacopoeia of the people's Republic of China. 1st Edn. Beijing: Beijing Chemical Industry Press, 223, 861–1102.409

[B29] Niu X. Xing W. Li W. Fan T. Hu H. Li Y. (2012). Isofraxidin exhibited anti-inflammatory effects *in vivo* and inhibited TNF-α production in LPS-induced mouse peritoneal macrophages *in vitro* via the MAPK pathway. Int. Immunopharm. 14 (2), 167–171. 10.1016/j.intimp.2012.06.022 22800929

[B30] Orlando B. J. Malkowski M. G. (2016). Substrate-selective inhibition of cyclooxygeanse-2 by fenamic acid derivatives is dependent on peroxide tone. J. Biol. Chem. 291, 15069–15081. 10.1074/jbc.M116.725713 27226593 PMC4946924

[B31] Rebetz J. Semple J. W. (2018). The pathogenic involvement of neutrophils in acute respiratory distress syndrome and transfusion-related acute lung injury. Transfus. Med. Hemotherapy 45, 290–298. 10.1159/000492950 PMC6257140 30498407

[B32] Shan J. Zhao Z. Fu J. Kong X. Huang H. Luo L. (2009). Chlorogenic acid inhibits lipopolysaccharide-induced cyclooxygenase-2 expression in RAW264.7 cells through suppressing NF-κB and JNK/AP-1 activation. Int. Immunopharmacol. 10.1016/j.intimp.2009.04.011 19393773

[B33] Shu J. Li L. Zhou M. Yu J. Ping C. Shao F. (2018). Three new flavonoid glycosides from Smilax glabra and their anti-inflammatory activity. Nat. Prod. Res. 32, 1760–1768. 10.1080/14786419.2017.1402314 29149807

[B34] Teng H. Chen L. (2018).Polyphenols and bioavailability: an update. Nutr. Crit. Rev. Food 52 (13), 2039–2051. 10.1080/10408398.2018.1437023 29405736

[B35] Teng H. Huang Q. Chen L. (2016). Inhibition of cell proliferation and triggering of apoptosis by agrimonolide through MAP kinase (ERK and p38) pathways in human gastric cancer AGS cells. Food Func. 7, 4605–4613. 10.1039/c6fo00715e 27747355

[B36] Tsai Y. C. Chen S. H. Lin L. C. (2017). Anti-inflammatory principles from Sarcandra glabra. J. Agric. Food Chem. 65, 6497–6505. 10.1021/acs.jafc.6b05125 28110531

[B37] Tsai Y. F. Chu T. C. Chang W. Y. Wu Y. C. Chang F. R. Yang S. C. (2017). 6-Hydroxy-5,7-dimethoxy-flavone suppresses the neutrophil respiratory burst via selective PDE4 inhibition to ameliorate acute lung injury. Free Radic. Biol. Med. 106, 379–392. 10.1016/j.freeradbiomed.2017.03.002 28263828

[B38] van de Veerdonk F. L. Netea M. G. van Deuren M. van de Meer J. W. de Mast Q. Brüggemann R. J. (2020). Kallikrein-kinin blockade in patients with COVID-19 to prevent acute respiratory distress syndrome. Elife 9, e57555. 10.7554/eLife.57555 32338605 PMC7213974

[B39] Wolter M. de Vink P. Neves J. F. Srdanovic S. Higuchi Y. Kato N. (2020). Selectivity via cooperativity: preferential stabilization of the p65/14-3-3 interaction with semisynthetic natural products. J. Am. Chem. Soc. 142, 11772–11783. 10.1021/jacs.0c02151 32501683 PMC8022324

[B40] Wright G. S. Antonyuk S. V. Kershaw N. M. Strange R. W. Samar Hasnain S. (2013). Ligand binding and aggregation of pathogenic SOD1. Nat. Commun. 4, 1758. 10.1038/ncomms2750 23612299 PMC3644087

[B41] Wu C. C. Chang S. C. Zeng G. Y. Chu H. W. Huang Y. Liu H. P. (2019). Proteome analyses reveal positive association of COL2A1, MPO, TYMS, and IGFBP5 with canine mammary gland malignancy. Proteonomics Clin. Appl. 13, e1800151. 10.1002/prca.201800151 30578659

[B42] Xiong Li. Zhao J. Liu J. Geng L. Zhao* Y. Zeng X. (2016). Systematic analysis of absorbed anti-inflammatory constituents and metabolites of Sarcandra glabra in rat plasma using ultra-high-pressure liquid chromatography coupled with linear trap quadrupole Orbitrap mass spectrometry. PloS One 11 (3), e0150063. 10.1371/journal.pone.0150063 26974321 PMC4790918

[B43] Yang J. Chen Y. Jiang K. Yang Y. Zhao G. Guo S. (2019). MicroRNA-106a provides negative feedback regulation in lipopolysaccharide-induced inflammation by targeting TLR4. Int. J. Biol. Sci. 15, 2308–2319. 10.7150/ijbs.33432 31595149 PMC6775322

[B44] Zhou M. Fang H. Du M. Li C. Tang R. Liu H. (2019). The modulation of regulatory T cells via HMGB1/PTEN/β-Catenin Axis in LPS induced acute lung injury. Front. Immunol. 10, 1612. 10.3389/fimmu.2019.01612 31402909 PMC6669370

[B45] Zielińska K. A. de Cauwer L. Knoops S. Molen K. V. Sneyers A. Thommis J. (2017). NK65 in combination with IFN-γ induces endothelial glucocorticoid resistance sustained activation of p38 and JNK. Front. Immunol. 8, 1199. 10.3389/fimmu.2017.01199 29033931 PMC5625030

