# Corrigendum: Combination effect of three main constituents from *Sarcandra glabra* inhibits oxidative stress in the mice following acute lung injury: a role of MAPK-NF-κB pathway

**DOI:** 10.3389/fphar.2023.1335005

**Published:** 2024-03-21

**Authors:** Chunping Liu, Jian-Xing Liu, Jiangyong Gu, Fang Liu, Jin Hua Li, Bin Yang, Yuan Zheng, Jie Li, Shou-Hai Wu, Qing-He Wu, Xian Zhang, Long-Mei Li, Hai-Long Yang, Lei Wang, Xiong Li

**Affiliations:** ^1^ The Second Affiliated Hospital of Guangzhou University of Chinese Medicine, Guangzhou, China; ^2^ Dongguan and Guangzhou University of Chinese Medicine Cooperative Academy of Mathematical Engineering for Chinese Medicine, Dongguan, China; ^3^ Research Center of Integrative Medicine, School of Basic Medical Science, Guangzhou University of Chinese Medicine, Guangzhou, China; ^4^ Institute of Tropical Medicine, Science and Technology Innovation Center, Guangzhou University of Chinese Medicine, Guangzhou, China; ^5^ Guangzhou Medical University School of Basic Medicine, Guangzhou, China

**Keywords:** *Sarcandra glabra*, chlorogenic acid, rosmarinic acid, isofraxidin, acute lung injury, MAPK-NF-kB

In the published article, there was an error in Figures 7B, C as published. The p38 strips in Figures 7B, C is a duplicate, which was incorrectly pasted during data processing.

**FIGURE 7:**
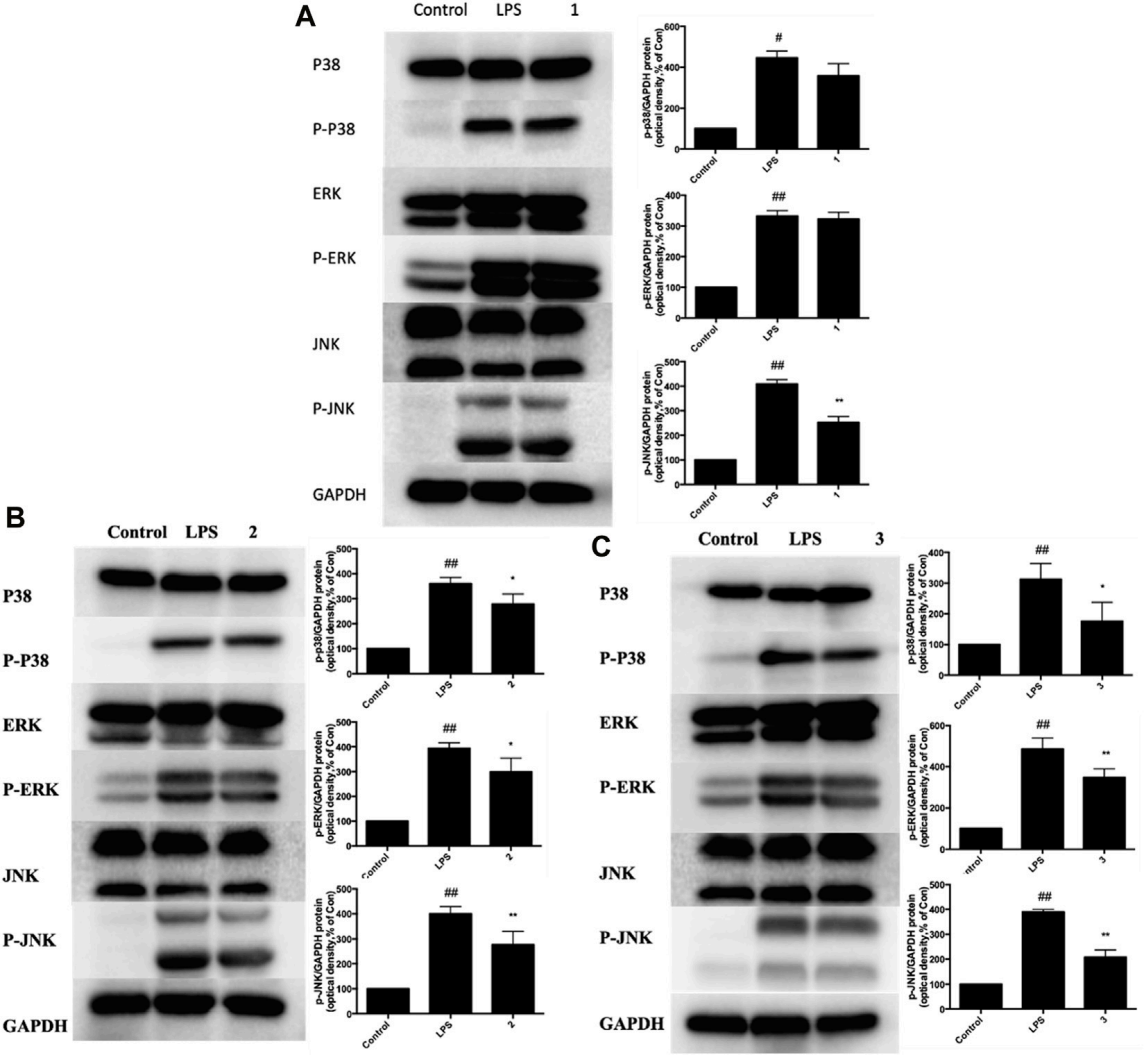
Effects of C, R, I (1, 2, 3) on p38 MAPK, ERK, and JNK activation in RAW264.7 cells stimulated by LPS. Pretreatment with; **(A)** C; **(B)** R; **(C)** I; Values are indicated as the mean of four mice per group. *p < 0.05, **p < 0.01 as compared to the LPS group, #*p* < 0.05, ##*p* < 0.01 as compared to the normal control group. Combined effect of C + R + I on inhibiting the activation of MAPK-NF-κB signaling pathway.

The corrected Figures 7B, C and its caption Effects of C, R, I (1, 2, 3) on p38 MAPK, ERK, and JNK activation in RAW264.7 cells stimulated by LPS appear below.

The authors apologize for this error and state that this does not change the scientific conclusions of the article in any way. The original article has been updated.

